# Alveolar defects before and after surgically assisted rapid palatal expansion (SARPE): a CBCT assessment

**DOI:** 10.1590/2177-6709.27.2.e2219299.oar

**Published:** 2022-06-10

**Authors:** Fábio Lourenço ROMANO, Cássio Edward SVERZUT, Alexandre Elias TRIVELLATO, Maria Conceição Pereira SARAIVA, Tung Tahan NGUYEN

**Affiliations:** 1 Universidade de São Paulo, Faculdade de Odontologia de Ribeirão Preto, Departamento de Clínica Infantil, área de Ortodontia (Ribeirão Preto/SP, Brazil).; 2 Universidade de São Paulo, Faculdade de Odontologia de Ribeirão Preto, Departamento de Cirurgia e Traumatologia Buco-Maxilo-Facial e Periodontia, área de Cirurgia (Ribeirão Preto/SP, Brazil).; 3 Universidade de São Paulo, Faculdade de Odontologia de Ribeirão Preto, Departamento de Clínica Infantil, área de Epidemiologia (Ribeirão Preto/SP, Brazil).; 4 University of North Carolina, School of Dentistry, Department of Orthodontics (Chapel Hill, USA).

**Keywords:** Palatal expansion, Surgically assisted rapid palatal expansion, Cone beam computed tomography, Orthodontics

## Abstract

**Introduction:**

Surgically Assisted Rapid Palatal Expansion (SARPE) promote maxillary expansion in skeletally mature patients. This technique is effective; however, some side effects are still unknown.

**Objectives:**

evaluate the presence of alveolar defects (dehiscences and fenestrations) in patients submitted to the SARPE. The null hypothesis tested was: SARPE does not influence the number of dehiscences and fenestrationss.

**Methods:**

A retrospective quasi-experiment study of a convenience sample of 279 maxillary teeth, in 29 patients evaluated with Cone Beam Computed Tomography (CBCT) at T1 (before SARPE), T2 (after expansion) and T3 (after retention), was performed. The examined teeth were: canines, first and second premolars, first and second molars. in axial, coronal, and cross-sectional views. The evaluations involved viewing slices from mesial to distal of the buccal roots.

**Results:**

All statistical analyses were performed using SAS 9.3 and SUDAAN softwares. Alpha used in the study was 0.05. Alveolar defects increased statistically from T1 (69.0%) to T2 (96.5%) and T3 (100%). Dehiscences increased 195% (Relative Risk 2.95) at the end of expansion (T2). After retention (T3), individuals were on average 4.34 times more likely to develop dehiscences (334% increase). Fenestrations did not increase from T1 to T2 (p = 0.0162, 7.9%) and decreased from T2 to T3 (p = 0.0259, 4.3%). Presence of fenestrations at T1 was a significant predictor for the development of dehiscences in T2 and T3. Dehiscences increased significantly in all teeth, except second molars.

**Conclusion:**

The null hypothesis was rejected. After SARPE the number of dehiscences increased and fenestrations decreased. Previous alveolar defects were predictor for dehiscences after SARPE.

## RESUMO

**Introdução:**

A expansão rápida da maxila assistida cirurgicamente (ERMAC) promove expansão em pacientes esqueleticamente maduros. Essa técnica é efetiva; entretanto, alguns efeitos colaterais ainda são desconhecidos.

**Objetivos:**

Avaliar a presença de defeitos alveolares (deiscência e fenestração) em pacientes submetidos à ERMAC. A hipótese nula testada foi que a ERMAC não influenciaria o número de deiscências e fenestrações.

**Métodos:**

Foi realizado um estudo quase-experimental de uma amostra de conveniência de 279 dentes superiores, de 29 pacientes que foram avaliados por meio de tomografia computadorizada de feixe cônico (TCFC) em T_1_ (antes da ERMAC), T_2_ (após expansão) e T_3_ (após contenção). Caninos, primeiros e segundos pré-molares, primeiros e segundos molares foram examinados em cortes axiais, coronais e sagitais. As raízes vestibulares desses dentes foram avaliadas da face mesial até a distal.

**Resultados:**

Todas as análises estatísticas foram realizadas usando os *softwares* SAS 9.3 e SUDAAN. O alfa usado no estudo foi de 0,05. Os defeitos alveolares aumentaram significativamente de T_1_ (69,0%) para T_2_ (96,5%) e T_3_ (100%). Deiscências aumentaram 195% (risco relativo de 2,95%) no final da expansão (T_2_). Após contenção (T_3_), os pacientes tiveram, em média, 4,34 vezes mais chance de desenvolver deiscência (334% de aumento). As fenestrações não aumentaram de T_1_ para T_2_ (*p*= 0.0162, 7.9%) e diminuíram de T_2_ para T_3_ (*p* = 0.0259, 4,3%). A presença de fenestrações em T_1_ foi um preditor significativo para o desenvolvimento de deiscências em T_2_ e T_3_. Deiscências aumentaram significativamente em todos os dentes, exceto nos segundos molares.

**Conclusão:**

A hipótese nula foi rejeitada. Após a ERMAC, o número de deiscências aumentou e o de fenestrações diminuiu. Defeitos alveolares prévios foram preditores de deiscências após a ERMAC.

## INTRODUCTION

Fenestrations and dehiscences are found in patients without orthodontic treatment.^1^ Many studies have examined the prevalence of alveolar defects during orthodontic treatment.^2^ ^-^ ^6^ Enhos et al.^7^ showed that there is more dehiscences in hyperdivergent than hypodivergent patients. However, there are conflicting results when the type of malocclusion (Class I or III) is evaluated.^6^ In general, dehiscences are more frequent in the mandible and fenestrations in the maxilla.^1,6,7^

Surgically Assisted Rapid Palatal Expansion (SARPE) is indicated for skeletally mature patients with severe maxillary transverse deficiency, with crowding, with a wide buccal corridor and failure of conventional maxillary expansion (RPE).^8^ This surgical orthodontic treatment evolved from cuts in maxillary resistance areas to LeFort I osteotomy with or without pterygoid disjunction to decrease pressure against the teeth that would affect the cortical bone and prevent periodontal defects.^9^ ^,^ ^10^ Orthodontic movements occur in the anchored teeth when the expander is activated after SARPE and can cause periodontal alterations in the bone.^5^ The long-term risks of having these alveolar defects and the problems associated with their increase post-SARPE should be addressed. The question is: has this worked to reduce the number of bone defects with the modern techniques and appliances? There is no consensus regarding the best orthodontic appliance to be used with surgical procedure nor the surgical techniques.^9^ ^-^ ^12^ Alterations promoted by SARPE have been evaluated in retrospective clinical studies using cephalometric radiographs, plaster models and CBCT imaging.^11^ ^,^ ^13^ ^-^ ^18^ However, the prevalence of fenestrations and dehiscences in patients that received SARPE still unknown. 

Thus, the aim of this study was to evaluate the presence of alveolar defects (dehiscences and fenestrations) in patients submitted to the SARPE. The null hypothesis tested was that the SARPE does not influence the number of dehiscences and/or fenestrations.

## MATERIAL AND METHODS

**Figure 1:**
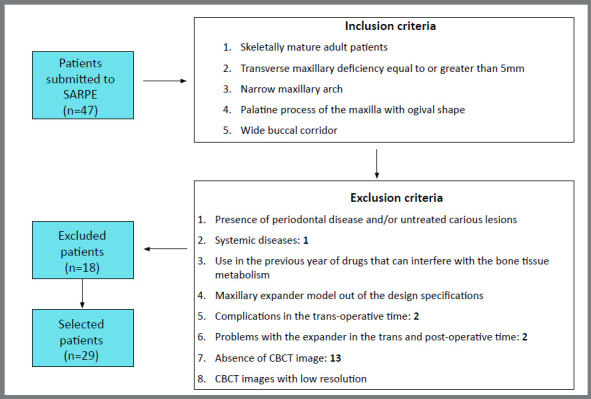
Study design ( Inclusion and exclusion criteria ).

The inclusion and exclusion criteria are presented in Figure 1. This study was approved by the Institutional Review Board of *Faculdade de Odontologia de Ribeirão Preto, Universidade de São Paulo* (#56387616600005419). Forty-seven consecutive adult patients from 2009 until 2014 were screened. All patients selected had Cone Beam Computed Tomography (CBCT) scans before the expansion procedure (T_1_), after expansion (T_2_) and at the end of the retention phase (T_3_). CBCT were requested by the surgeon for surgical evaluation before and after SARPE. After exclusion criteria was applied, a total of 29 patients (mean age = 30.4 years; 17.2 - 54.11) participated in the study (16 males, 13 females). This study was a before and after retrospective quasi-experiment of a convenience sample, however the number of patients select was similar to other articles that evaluated SARPE.^8^ ^,^ ^11^ ^,^ ^14^

**Figure 2:**
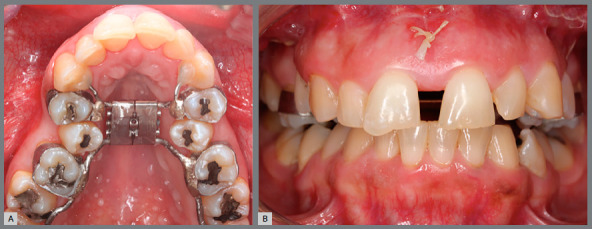
A) Hyrax expander with bands on the first molars and first premolars. Patients were instructed to perform two turns in the morning and two turns at night. B) Overcorrection of maxillomandibular transverse relationship.

**Figure 3:**
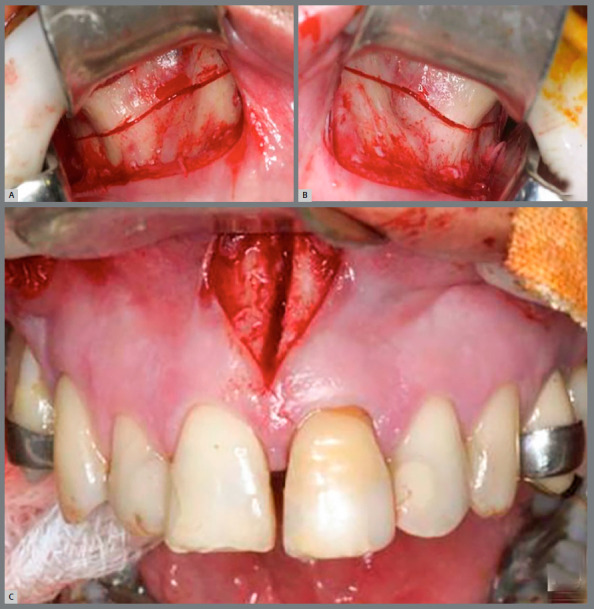
Conventional bilateral LeFort I osteotomy and midline separation: A) right side; B) left side; C) anterior view.

A Hyrax expander with bands on the first molars and first premolars, and extensions arms to canines and second molars was fabricated (Fig 2A). The surgeries were performed by three experienced oral maxillofacial surgeons. The surgical technique consisted of conventional bilateral LeFort I osteotomy and midline separation between central incisors. Pterygoid disjunction and down fracture were not performed (Fig 3). Three oral maxillofacial surgeons performed the same SARPE surgical protocol in all patients under general anesthesia at a public hospital in Ribeirão Preto, Brazil. The patients received nasotracheal intubation while lying in the supine position. According to the hospital protocol, 2 g of cefazolin sodium and 10 mg of dexamethasone were administered intravenously as antibiotic and antiemetic prophylaxis.

A topical solution of 10% povidone-iodine was used for intraoral and facial sterilization. After the placement of sterile surgical drapes and oropharyngeal protection, 6 mL of an anesthetic solution of 2% lidocaine with 1:100,000 epinephrine was administered along the buccal sulcus of the maxilla. Using a #15 blade mounted on a #3 scalpel handle, an incision was made in the buccal sulcus of the maxilla (vestibule) at about 4 mm from the mucogingival junction, extending from the mesial surface of the first molar to the mesial surface of the canine. After mucoperiosteal flap elevation, the surface of the maxillary bone was exposed from the zygomatic buttress to the most inferolateral point of the piriform aperture. Osteotomy was performed from this point to a slightly posterior point in the zygomatic buttress, 5 mm above the roots of the posterior teeth, using a #702 taper fissure crosscut carbide bur under abundant saline irrigation. The same procedure was performed in the other side. An incision was made in the maxillary labial frenum sagittally, and an osteotomy was performed using a #701 taper fissure crosscut carbide bur under abundant saline irrigation after mucoperiosteal flap elevation and placement of a retractor (Minnesota-Sverzut; Quinelato, Rio Claro, Brazil).

The osteotomy was performed following the intermaxillary suture and was completed using a chisel and mallet (Sverzut; Quinelato, Rio Claro, Brazil). After the fracture was made, the expansion screw was activated 2 mm (eight ¼ turns) then deactivated 1 mm to open a 1 mm diastema between the maxillary central incisors. 

Seven days after surgery, the patients began to turn the screw with two 1/4 turns in the morning and two 1/4 turns at night. Expansion was stopped when the palatal cusps of the maxillary molars occluded with the buccal cusps of the mandibular molars (Fig 2B) and held in retention for six months.

CBCT were requested by the oral and maxillofacial surgeon to evaluate the surgical aspects of SARPE. CBCT scans were obtained with an i-CAT scanner (International Imaging Sciences, Hatfield, PA) using 120kV, 5mA, 0.4 mm voxel, with 40s scanning and 16x22 cm field of view. The patients were seated in an upright position with the Frankfort plane parallel to the floor and in centric occlusion. CBCTs were initially oriented to Frankfort-horizontal and readjusted to the 3D occlusal plane. This was done to have the coronal plane as parallel as possible to the long axis of the teeth. CBCT DICOM (Digital Imaging and Communications in Medicine) files were first converted to gipl format, and multiplanar slices were viewed using ITK-SNAP 3.4 (http://www.itksnap.org). 

**Figure 4:**
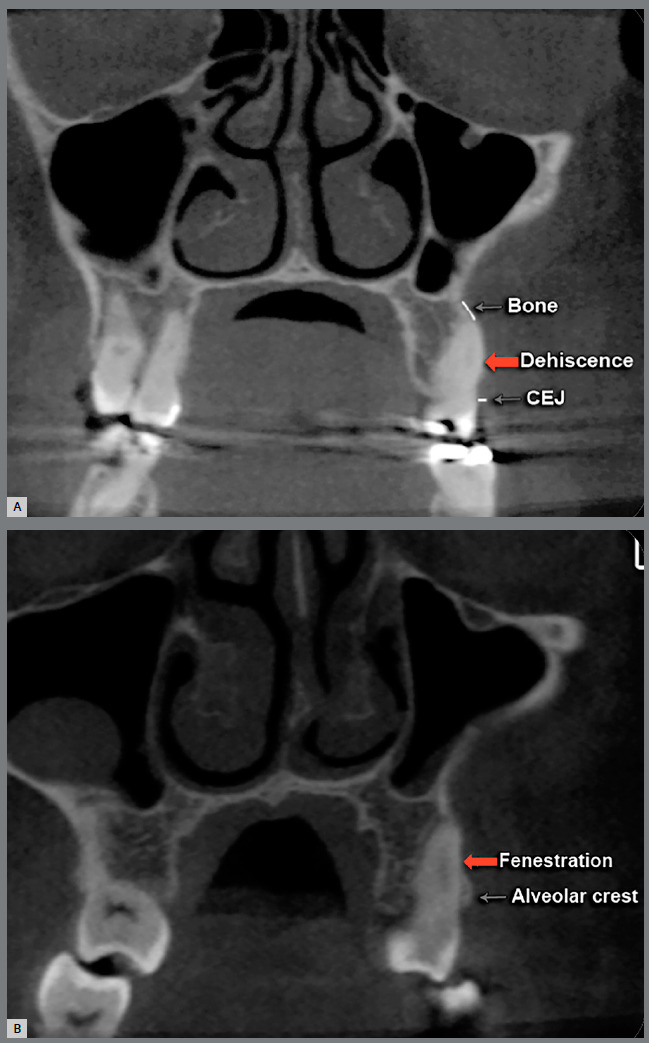
CBCT coronal slice showing dehiscence **(**A) and fenestration **(**B).

A total of 1,170 buccal sites in 279 teeth (canines, first and second premolars, first and second molars) in both sides of the maxilla were evaluated for dehiscences and fenestrations at T_1_, T_2_ and T_3_. Some patients had missing teeth (total of 11 teeth). Distal and mesial roots were evaluated separately for the molars, however at the same time. Evaluations was primarily conducted on the coronal view, but all three planes were carefully examined using the multi-planar view. Furthermore, each individually tooth was evaluated slice by slice from the mesial portion to the distal portion of the root, so the diagnosis of dehiscences and fenestrations was not done solely using a single coronal slice but confirmed through multiple slices. When the alveolar bone height was more than 2 mm from the cementoenamel junction (CEJ), it was classified as dehiscence (Fig 4A). If the bone defect did not involve the alveolar crest, it was classified as fenestrations (Fig 4B).^1^ ^,^ ^6^ ^,^ ^7^ Evaluations were performed in dark room to improve the visualization of alveolar defects,^6^ in a 27-in retina 5k display with high resolution, by a calibrated orthodontist and independently evaluated by another orthodontist, with high interexaminers agreement. These different evaluations were blinded. After two weeks, all measurements were re-analyzed by one examiner, to determine the reproducibility of the method.

### STATISTICAL ANALYSIS

Kappa coefficient assessed the reproducibility of the method. Baseline comparisons of presence of defects (no defect, one of the defects or both) among males and females were performed using exact tests for proportions. Age differences for presence of defects were tested using Kruskall-Wallis test, because age was not normally distributed. Distribution of defects for each time was evaluated considering dependence of observations within individual and within tooth, by means of Wald chi-square. Relative Risks (crude and adjusted) with 95% Confidence Intervals for development of dehiscence (α= 0.05) were estimated using Poisson regression with robust estimators. Risk modeling was estimated considering cluster of individuals and teeth controlling for side (right/left), type of teeth (first molar, second molar, premolar, and canine), age and sex. Final model included not only significant variables, but also those who could be potential confounders, such as age and sex. All statistical analyses were performed using SAS 9.3 (SAS Institute Inc, Cary, NC) and SUDAAN callable (RTI International, Cary, NC).

## RESULTS

At T_1_, 69.0% of the patients exhibited some type of alveolar defects. The distribution of one or two alveolar defects was not different (*p* = 0.7212) among males (25.0% and 37.5%) and females (23.1% and 53.8%). Median age of participants was higher (*p* = 0.0276) for those with one bone defect (44.2 years, Q1 = 35.6 - Q3 = 52.8) compared to those with none (25.8 years; Q1 = 21.4 - Q3 = 29.0) or two bone defects (25.0 years; Q1 = 22.5 - Q3 = 28.2). 

Alveolar defects increased to 96.5% at T_2_ and 100% at T_3_. There was an 18.9% increase in the number of surfaces exhibiting dehiscences from T_1_ to T_2_ and 13.5% increase from T_2_ to T_3_. On the other hand, the number of surfaces with fenestrations decreased 3.6% from T_2_ to T_3_. With treatment all teeth but second molars showed statistically significant increase in dehiscences development (*p* = 0.2263). The most affected teeth were first molars, followed by the first premolars (Table 1).

**Table 1:** Fenestration and dehiscence per tooth at each time point.

	T1	T2	T3	p*
	n (%)	n (%)	n (%)	
Second molar				
No defect	105 (98.2)	101 (94.4)	97 (90.7)	0.2263
Fenestration	1 (0.9)	3 (2.8)	5 (4.6)	
Dehiscence	1 (0.9)	3 (2.8)	5 (4.6)	
First molar				
No defect	70 (60.3)	35 (30.2)	18(15.2)	< 0.0001
Fenestration	21 (18.1)	17 (14.7)	4 (3.5)	
Dehiscence	25 (21.6)	64 (55.2)	94 (81.0)	
Second premolar				
No defect	54 (93.0)	43 (74.1)	38 (65.2)	0.0054
Fenestration	2 (3.5)	4 (6.9)	4 (6.9)	
Dehiscence	2(3.5)	11 (19.0)	16 (27.6)	
First premolar				
No defect	38 (73.1)	19 (36.5)	8 (15.4)	< 0.0001
Fenestration	6 (11.5)	6 (11.5)	2 (3.8)	
Dehiscence	8 (15.4)	27 (51.9)	42 (80.8)	
Canine				
No defect	56 (96.5)	51 (87.9)	49 (84.5)	< 0.0001
Fenestration	0 (0.0)	0 (0.0)	1 (1.7)	
Dehiscence	2 (3.5)	7 (12.1)	8 (13.8)	

*p-value for Wald chi-square of association between time and defects, taking into consideration dependence of observation within individual and tooth.

Analysis of the risk for development of dehiscences (Table 2) showed an increase of 2.95 at T_2_ and 3.34 at T_3_ compared to T_1_. Age, sex, and side were not associated with the development of dehiscences. The first molars were 3.71 times more likely to develop dehiscences. The presence of alveolar defects at the T1 was a significant predictor for the development of new dehiscences. Surfaces with fenestrations at T_1_ showed a higher likelihood of developing dehiscences at T_3_. High Kappa coefficient values for intraobserver agreement were found: 0.99 for dehiscences and 1.00 for fenestrations.

**Table 2:** Comparison of the probability for the increase in the number of dehiscence between the study times, sides, teeth, surfaces, sex and age.

	RR crude	95% CI	p	RR adjusted	95% CI*	p†
Time						
T1	1			1		< 0.0001
T2	2.95	2.31 - 3.77	< 0.0001	2.95	2.31 - 3.77	
T3	4.34	3.35 - 5.63		4.34	3.35 - 5.63	
Side						
Right	1		0.4356	1		0.1353
Left	1.13	0.83 - 1.54		1.16	0.94 - 1.41	
Teeth						
Second molar	0.17	0.05 - 0.55		0.14	0.04 - 0.44	< 0.0001
First molar	3.16	1.99 - 5.00	< 0.0001	2.6	1.61 - 4.19	
Second premolar (ref)	1	1		1	1	
First premolar	2.96	1.84 - 4.77		3.02	1.87 - 4.89	
Canine	0.59	0.26 - 1.32		0.59	0.26 - 1.31	
Surfaces						
Distal	1			1		
Mesial	1.43	1.16 - 1.77	0.002	1.43	1.16 - 1.77	0.001
Unique face	0.73	0.75 - 1.44		1	1.00 - 1.00	
Sex						
Male	1			1		0.0684
Female	0.98	0.72 - 1.33	0.8931	0.83	0.67 - 1.01	
Age (years)						
≤ 35	1			1		0.2487
> 35	1.11	0.79 - 1.56	0.5424	1.26	0.85 - 1.89	
Presence of defects at baseline						
No defect	1		0.0555	1		0.0003
One defect	1.23	0.80 - 1.89	0.0168_(trend)_	1.06	0.69 - 1.63	
Both defects	1.52	1.08 - 2.15		1.58	1.25 - 2.01	

* 95% CI = 95% confidence interval. † p-value for Wald chi-square statistics. On the left side, is the crude RR with 95% confidence Intervals; and on the right side, the adjusted RR. Each RR should be interpreted as the RR independent of the other variables in the model, even though sex, age and side were not significant in the model.

## DISCUSSION

It is estimated that 33.44% of Class I patients and 23.84% of Class II, division 1 without prior orthodontic treatment show some type of alveolar defect.^6^ In the present study, 69.0% of patients had alveolar defects before treatment, increasing to 96.5% after expansion and 100.0% in the retention phase. This increase was not surprising since most of the patients had dehiscences and fenestrations prior to SARPE. However, the increase of dehiscences was very large and occurred over a short time period, indicating the likelihood of side effects created by the expansion. However, confounding effects, as deficient hygiene, may have contributed to this. It is worth mentioning that some authors agreed that fenestrations prevalence is overestimated in CBCT scans.^21^ ^,^ ^22^ Although it was not the objective of this study, the thickness of the vestibular alveolar bone may influence the amount of post-expansion alveolar defects.^11^ Before SARPE, 20% of the teeth had alveolar defects. This is less than the data reported by Enhos et al.^7^ (33.53%), however, their study included mandibular incisors, which shows a higher percentage of alveolar defects.^1^ ^,^ ^6^ ^,^ ^7^ In this study, correlation among malocclusion, growth pattern with number of dehiscences and fenestrations was not evaluated. Further studies are needed to test that hypothesis. 

**Figure 5:**
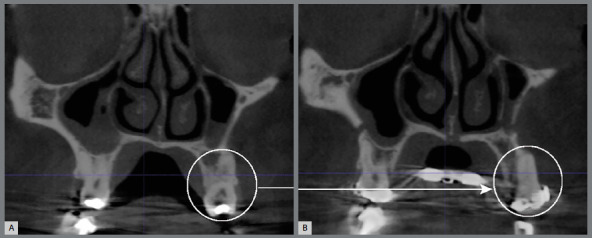
Fenestration (**A**) of the maxillary first molar (mesial root) changed, in the same patient, to dehiscence (**B**) after SARPE.

Buccal movements of the teeth can lead to dehiscences.^18^ The present data showed increase in dehiscences between T1-T2, T2-T3 and T1-T3 of 18.9%, 13.5% and 32.4%, respectively. Periodontal evaluation in adult patients before orthodontic treatment is essential to determine alveolar morphology and to prevent undesirable effects.^6^ ^,^ ^7^ Clinical implication is that patients with alveolar defects before SARPE have tendency to develop more dehiscences after treatment. This information is clinically relevant, and it may help orthodontists and surgeons predict and try to control complications. Perhaps performing a pterygoid disjunction during the SARPE procedure may reduce posterior bony resistance and thereby decrease the number of dehiscences after expansion. However, studies reported that the width of buccal alveolar bone decreased for all posterior teeth after SARPE, regardless of surgical technique (with or no pterygoid disjunction).^19^ It is possible that this bone decrease might be due to the tilting of maxillary bone.^20^ In the present study, the prevalence of fenestrations decreased from T_2_ to T_3_. This is because many fenestrations became dehiscences in T_2_ or T_3_ due to uncontrolled teeth tipping (Figs 4 and 5).

Studies^21^ ^-^ ^23^ validated the accuracy and reliability of the CBCT in the diagnosis of alveolar defects for different voxel sizes (0.2 mm to 0.4 mm). It is important to note that radiograph may differ from clinical outcomes. Significant decrease in buccal alveolar bone thickness following SARPE has been reported despite few side effects on the periodontium in clinical examination.^11^ ^,^ ^24^ However, it is possible that the clinical impact in periodontal health may take longer to occur, due to adaptation of soft and hard tissues.

Of the 279 teeth examined at each study time, 35.4% had of alveolar defects in the three time points of the present study. The alveolar defects significantly increased in all tooth type, except second molar. The teeth with greater alveolar defects were first molar (64.6%) and first premolar (57.2%), probably because they were banded, receiving more expansion forces. Conversely, in two other studies^1^ ^,^ ^6^ the canines were among the teeth more affected by alveolar defects. However, it is important to point out the difficulty in measuring the alveolar bone of canines, due to their location in the corner of the mouth.

Although SARPE had been extensively evaluated, some doubts remain about this surgical-orthodontic technique. Is there relationship between the velocity of expansion and the development of alveolar defects? Intuitively, this might sound correct, but the literature is controversial about the activation rate (ranges from 0.25 mm to 1.0 mm per day).^25^ In this study, the activation followed the osteogenic distraction protocol that is widely used bt orthodontics and surgeons^26^ ^,^ ^27^ and the activation initiated seven days after surgery. Perhaps, the time to initiate activation might have impacted the degree of expansion. It is hypothesized that early activation (two days post-operative) improves expansion because it limits bone and soft tissue healing, which could increase sutural resistance.^26^

Why in this study did the alveolar defects increase with time, considering that the expander remained in the mouth without activation during the retention phase? Oliveira et al.^26^ observed correction tendencies in root inclination of the first molar after removal of the expander. On the other hand, Gurgel et al.^27^ reported no dental inclination post-expansion, with appliance still in the mouth. It is possible that retaining the inclination of the crown and roots during the retention phase may have contributed to formation of new dehiscences. Probably there was continuation of expansion even after immobilization of the expander screw, and it caused increase of the amount of dehiscence from T_1_ to T_2_ in some patients.

There were limitations in this study since it was difficult to select patients with specifics characteristic (inclusion criteria) and with complete documentation. Evaluation of canines was imprecise because this tooth is in the corner of the mouth. In order to correctly visualize dehiscences and fenestrations in the canines, it was necessary to examine in coronal view and compare with cross-sectional view. Another limitation of the study was short evaluation time (six months). Some confounding factors were age variation of the sample and expansion amount. A long-term study could show whether dehiscences will continue after lingual tipping of the teeth and stability for a long time.^28^

Future studies should focus on association of SARPE with orthodontic treatments to correct skeletal malocclusions^29^, or if incorporating skeletal anchorage-borne expanders may achieve ideal transverse expansion while decreasing the likelihood of developing dehiscences. Finally, a thorough 3D evaluation of the periodontium of the patient is recommended, making it possible to plan adequate expansion and avoid side effects. It is also recommended an informed consent regarding the increased risk of developing alveolar defects before initiating SARPE.

## CONCLUSION

The null hypothesis was rejected. Dehiscences increased and fenestrations decreased after SARPE. First molars and first premolars had the greater number of alveolar defects. Presence of previous alveolar defects was predictor for dehiscences after SARPE.
